# Better or Worse? The Independent Prognostic Role of HPV-16 or HPV-18 Positivity in Patients With Cervical Cancer: A Meta-Analysis and Systematic Review

**DOI:** 10.3389/fonc.2020.01733

**Published:** 2020-10-07

**Authors:** Xing Chen, Ping Zhang, Shanshan Chen, Hanxiao Zhu, Kai Wang, Liya Ye, Jun Wang, Junhui Yu, Shuangshuang Mei, Zhengrong Wang, Xiaodong Cheng

**Affiliations:** ^1^Zhejiang Taizhou Hospital, Taizhou, China; ^2^Zhejiang Cancer Hospital, University of Chinese Academy of Sciences, Hangzhou, China; ^3^Women's Hospital, School of Medicine, Zhejiang University, Hangzhou, China

**Keywords:** human papillomavirus 16, human papillomavirus 18, uterine cervical neoplasms, prognosis, meta-analysis

## Abstract

**Background:** The literature reports conflicting results regarding the effect of human papillomavirus (HPV) genotype 16 (HPV-16)/18 (HPV-18) positivity on cervical cancer (CC) prognosis.

**Aim:** To conduct a meta-analysis to examine the effect of HPV-16/18 positivity on the prognosis of patients with CC.

**Methods:** PubMed, Embase, and the Cochrane Library were searched for available papers published up to March 2020. The main outcome was the hazard ratio (HR) of overall survival (OS) or disease-free survival (DFS) comparing HPV-16 or HPV-18 positivity and negativity. The random-effects model was used for synthesizing survival outcomes.

**Results:** Nine studies and 2,028 patients were included. Four studies reported OS in HPV-16 positivity, and no association was found between HPV-16 positivity and OS to CC (HR = 0.79, 95% CI: 0.26–2.39, *P* = 0.675). Three studies reported DFS in HPV-16 positivity, and no association was found between HPV-16 positivity and DFS to CC (HR = 0.80, 95% CI: 0.30–2.11, *P* = 0.654). Two studies reported DFS in HPV-18 positivity, and no association was found between HPV-18 positivity and DFS to CC (HR = 0.99, 95% CI: 0.55–1.78, *P* = 0.984). One study reported progression-free survival (PFS) in HPV-18 positivity, and an association was observed between HPV-18 positivity and PFS to CC (HR = 2.66, 95% CI: 1.44–4.94, *P* = 0.002). The sensitivity analyses showed that one study biased the analysis of the association between HPV-16 and OS, and another study biased the association between HPV-16 and DFS.

**Conclusion:** The presence of HPV-16 and HPV-18 positivity appears to have no significant association with prognosis in CC in either OS or PFS. The presence of HPV-16 or HPV-18 positivity has no significant association with prognosis in CC in either OS or PFS.

## Introduction

Cervical cancer (CC) is a malignancy originating in the transformation zone of the cervix, most commonly in squamous cells (1). It is the second most common cancer in women worldwide, with an estimated 569,847 new cases in 2018, and the third most common cause of female cancer mortality, with 311,365 deaths (2, 3). CC has a strong tendency to affect young women, and the peak incidence is in the 40–49 age group (3, 4).

Infection with high-risk human papillomavirus (HPV) is a major risk factor for the development of CC (1, 4–8). It is now well-recognized that the majority of CC is associated with HPV genotypes 16 (HPV-16) and 18 (HPV-18) (6, 9–11). HPV, an epitheliotropic double-stranded DNA oncovirus, typically infects the basal layer of the epithelium through small tears in the mucosa resulting from sexual activity. Active papillomavirus infection occurs when infected basal cells replicate and fill the area. HPV synthesizes six early proteins (E1–E7) and two late capsid proteins (L1 and L2) during replication, and those proteins have immortalizing and transforming properties (1). Persistent HPV infection results in squamous intraepithelial lesions that are graded as cervical intraepithelial neoplasia (CIN) 1, CIN 2, and CIN 3 according to how much epithelium is impacted. The progression from cervical dysplasia to invasive cancer may take years or decades but has been reported to take <1 year in about 10% of patients (12).

Nevertheless, despite the sound pathogenic effect of HPV-16 and HPV-18 for CC, the prognosis of HPV-16 and HPV-18 positivity in patients with CC has not been established. A recent meta-analysis revealed that HPV DNA positivity is associated with good overall survival (OS) and disease-free survival (DFS) in patients with CC (13). Similar meta-analyses reported that HPV positivity was an indicator of favorable prognosis in head and neck cancers (14, 15), but there are studies that failed to support the relationship between HPV-16 or HPV-18 positivity and prognosis of CC (16), and some even indicate that CC associated with HPV-16/18 has a worse survival (17).

Gaining a more comprehensive insight into how the HPV type affects survival is important. We herein hypothesized that HPV-16/18 positivity is associated with poorer prognosis in patients with CC. To test our hypothesis, we conducted this meta-analysis and systematically reviewed the existing literature.

## Methods

### Literature Search

This meta-analysis was conducted according to the Preferred Reporting Items for Systematic Reviews and Meta-Analyses (PRISMA) guidelines (18). The search was based on the PICO principle (19), followed by screening using a prespecified protocol and eligibility criteria: (1) population: patients with CC who had a record of HPV genotype; (2) exposure: HPV-16 or HPV-18 positivity; (3) controls: HPV-16 or HPV-18 negativity; (4) outcome: survival; and (5) full text available in English. PubMed, Embase, and the Cochrane Library were searched for available papers published up to March 2020 using the MeSH term “Uterine Cervical Neoplasms,” as well as relevant keywords.

### Data Extraction

The study characteristics (authors, year of publication, country where the study was performed, median follow-up time, sample size, and mean age in each group), treatment parameters [The International Federation of Gynecology and Obstetrics (FIGO) stage of CC, histology, detection method of HPV genotype, reported HPV genotype, the operation the patients underwent, the endpoint of the study, type of specimens that were used, and covariates if a multivariable model was used], and outcome (OS and DFS) were extracted by two authors independently. Any discrepancy was solved by discussion.

### Outcomes

The main outcome was the hazard ratio (HR) of OS or DFS comparing HPV-16 or HPV-18 positivity and negativity on DFS or OS.

### Quality of the Evidence

The quality level of evidence of all articles was assessed independently by two authors according to the Newcastle–Ottawa scale (NOS) for cohort study (20). Discrepancies in the assessment were resolved through discussion until a consensus was reached.

### Data Synthesis

The risk estimates of each study were reported as HR or relative risk (RR). We treated RRs as HRs. When possible, multi-adjusted HRs were used in the meta-analysis.

### Statistical Analysis

All analyses were performed using STATA SE 14.0 (StataCorp, College Station, Texas, USA). HRs and corresponding 95% confidence intervals (CIs) were used to compare the outcomes. Statistical heterogeneity among studies was calculated using Cochran's *Q*-test and the *I*^2^ index. An *I*^2^ > 50% and a *Q*-test *P* < 0.10 indicated high heterogeneity, and the random-effects model was used; otherwise, the fixed-effects model was applied. *P* < 0.05 were considered statistically different. We did not assess potential publication bias by funnel plots and Egger's test because the numbers of studies included in each quantitative analysis were <10, in which case, the funnel plots and Egger's test could yield misleading results (21).

## Results

### Selection of the Studies

**Figure 1:**
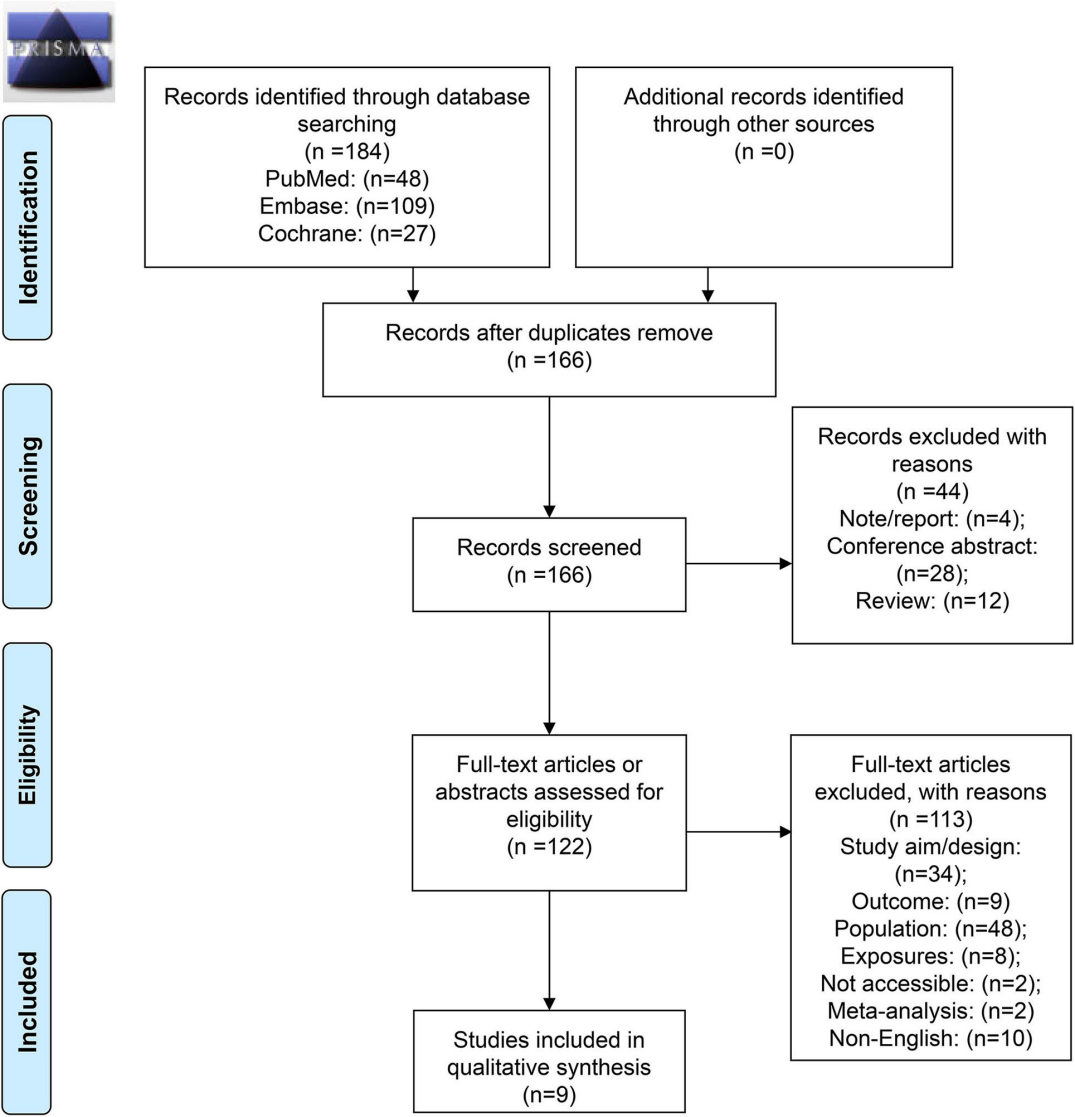
Preferred reporting items for systematic reviews and meta-analyses (PRISMA) 2009 flow diagram.

Figure 1 presents the selection flowchart. In the initial search, 184 records were retrieved, and 166 were screened after removing the duplicates. From them, 44 were excluded because of the publication type (notes, conference abstracts, and reviews). Then, 122 full-text papers were assessed and 113 were excluded because of study aim/design (*n* = 34), outcome (*n* = 9), population (*n* = 48), exposures (*n* = 8), full text not accessible (*n* = 2), meta-analyses (*n* = 2), and non-English (*n* = 10).

Therefore, nine studies were included (16, 17, 22–28) (Table 1). Those studies included a total of 2,028 patients. The mean age range was 47–57 years. The median follow-up ranged from 33 to 136 months. Four studies scored 7 on the NOS (24, 26–28), four studies scored 8 (16, 22, 23, 25), and one study scored 9 (17) (Supplementary Table 1).

**Table 1:** Literature search and study characteristic.

**Reference**	**Country**	**FIGO stage**	**Histology**	**Method of detecting HPV**	**HPV genotype reported**	**Surgical operation if mentioned**	***n***	**Age (Exposure/** **control)**	**Median follow-up**	**Endpoint**	**Type of specimens**	**Covariates in the model**
(22)	Korea	ALL	SCC, AC, ASC	PCR	HPV 16	Laparoscopic or robotic radical hysterectomy with pelvic and/or para-aortic lymphadenectomy	248	51.4 ± 11.5	59	DFS	Fresh	Age, FIGO stage, tumor size, lymph node metastasis
(16)	China	ALL	SCC, AC, ASC	PCR	HPV 16, HPV 18	Radical hysterectomy with pelvic lymphadenectomy	306	48 (26–71)	54	OS	fresh	Age, FIGO stage, treatment
(23)	Korea	IB–IIA	SCC, AC, ASC	PCR	HPV 18	Radical hysterectomy with pelvic lymphadenectomy	204	47.4 ± 11.8/ 49.5 ± 11.7	NR	PFS	Fresh	Histology, stage, tumor size, lymph node metastasis
(24)	Korea	IIA–IVB	SCC, AC, ASC	PCR	HPV 18	NR	181	57 (23–80)	33	DFS	paraffin	Age, stage nodal status, histologic grade, histologic type, tumor size, smoking
(25)	Russia	III	NR	PCR	HPV 16	Chemo- and radiotherapy or radiotherapy alone	92	NR	NR	DFS	fresh	NA
(26)	Taiwan	I–IV	AC, ASC	PCR	HPV 16	Primary definitive surgery	452	48.3 (27.5–89.2)	136	DFS	paraffin	Age, FIGO stage, grade
(27)	Japan	ALL	SCC, AC, ASC, SCCC	PCR	HPV 16	NR	137	49.2 ± 14.8	102.5	OS	fresh	Age, FIGO stage, histology
(17)	Germany	I–II	SCC, AC, ASC	PCR	HPV 16	Radical hysterectomy and pelvic lymphadenectomy	121	48.1	42	OS	Paraffin	Depth of invasion, tumor grade, node metastases, HPV in histologically confirmed cancer-free pelvic lymph nodes
(28)	Germany	I–IIa	SCC, AC, ASC, SCCC	SBH+PCR	HPV 16	Intracavitary radiotherapy, radical hysterectomy and pelvic lymphadenectomy	287	NR	NR	OS	fresh	Age, histology, hospital

*SCC, squamous cell carcinoma; AC, adenocarcinoma; ASC, adeno-adenosquamous carcinoma; SCCC, small cell carcinoma of the cervix; HPV, human papillomavirus; FIGO, The International Federation of Gynecology and Obstetrics; DFS, disease-free survival; OS, overall survival; PFS, progression-free survival*.

### Survival According to Human Papillomavirus Subtype 16

**Figure 2:**
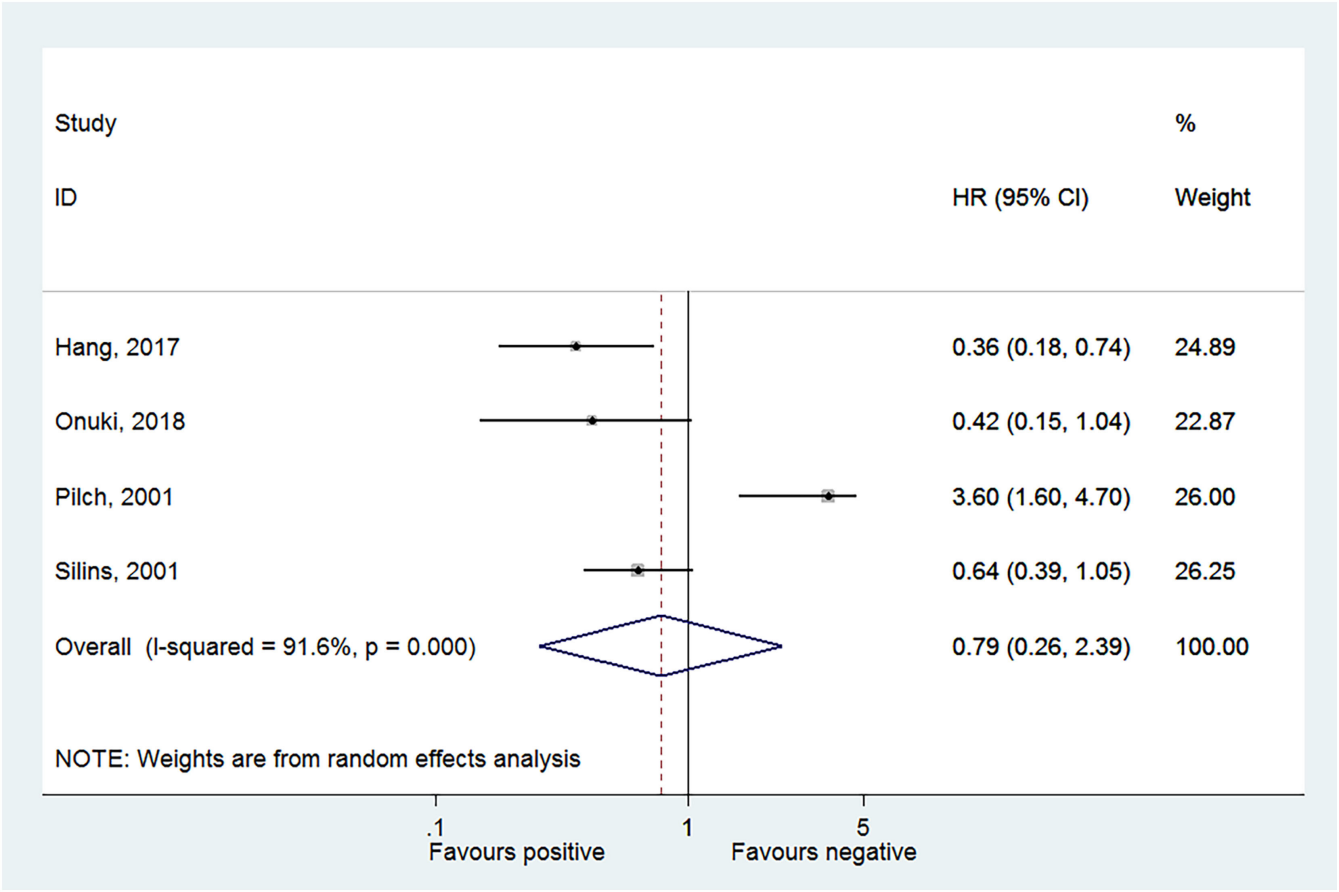
Forest plot of overall survival comparing the human papillomavirus subtype 16 (HPV-16) positive vs. negative groups.

**Figure 3:**
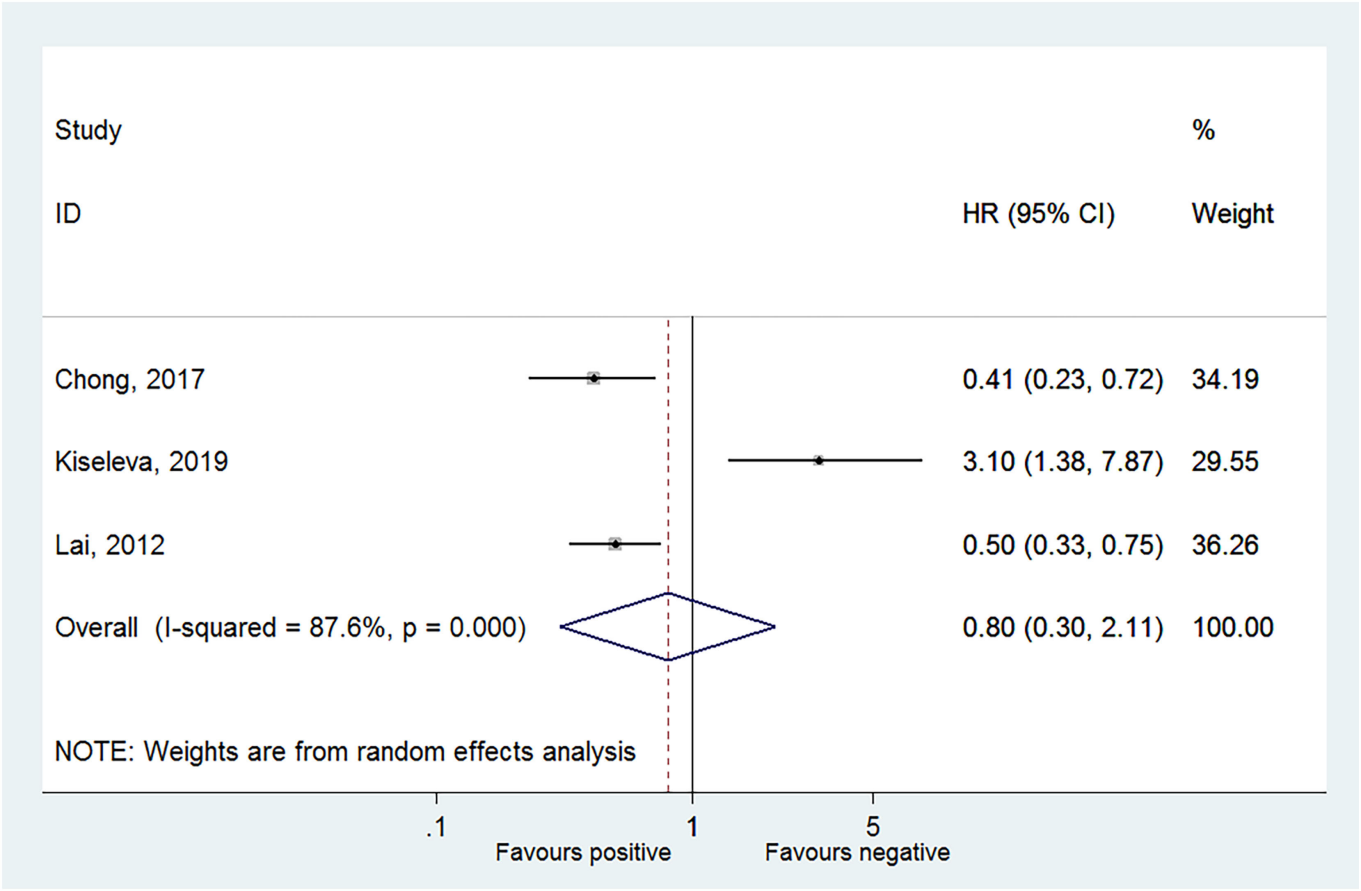
Forest plot of disease-free survival comparing the human papillomavirus subtype 16 (HPV-16) positive vs. negative groups.

Four studies reported OS in HPV-16 positivity (16, 17, 27, 28), and no association was found between HPV-16 positivity and OS to CC (HR = 0.79, 95% CI: 0.26–2.39, *P* = 0.675; *I*^2^ = 91.6%, *P*_heterogeneity_ < 0.001) (Figure 2, Table 2). Three studies reported DFS in HPV-16 positivity (22, 25, 26), and no association was found between HPV-16 positivity and DFS to CC (HR = 0.80, 95% CI: 0.30–2.11, *P* = 0.654; *I*^2^ = 87.6%, *P*_heterogeneity_ < 0.001; Figure 3, Table 2).

**Table 2:** Results from the meta-analysis. HPV genotype positive vs. negative.

	***N***	**HR (95% CI)**	***P***	***I*-square**	***P* (Heterogeneity)**
**HPV16**					
OS	4	0.79 (0.26, 2.39)	0.675	91.6	<0.001
DFS	3	0.80 (0.30, 2.11)	0.654	87.6	<0.001
**HPV18**					
DFS	2	0.99 (0.55, 1.78)	0.984	0.0	0.853
PFS	1	2.66 (1.44, 4.94)	0.002		

*HPV, human papillomavirus; DFS, disease-free survival; OS, overall survival; PFS, progression-free survival; HR, hazard ratio*.

### Survival According to Human Papillomavirus Subtype 18

**Figure 4:**
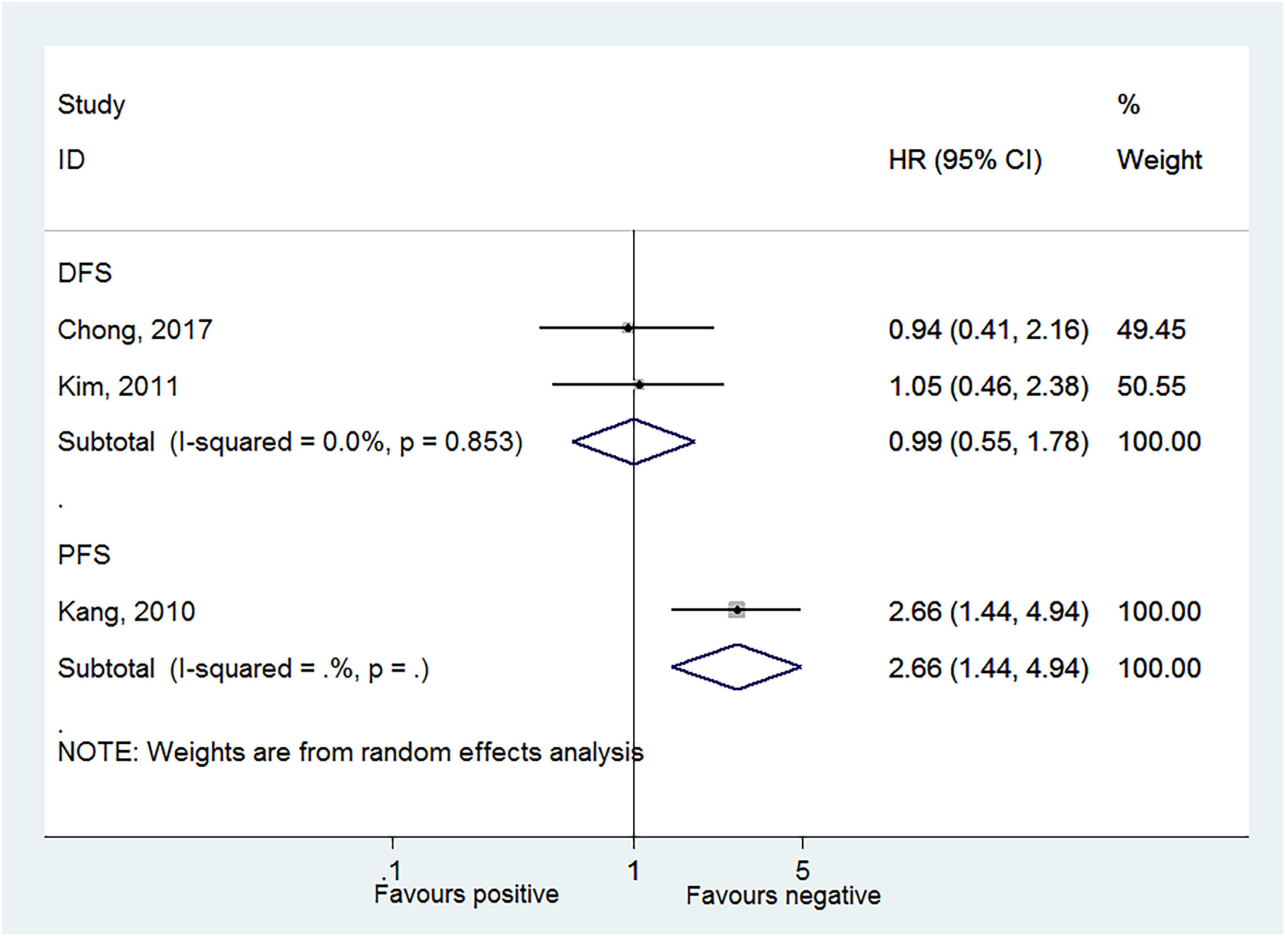
Forest plot of disease-free survival and progression-free survival comparing the human papillomavirus subtype 18 (HPV-18) positive vs. negative groups.

Two studies reported DFS in HPV-18 positivity (22, 24), and no association was found between HPV-18 positivity and DFS to CC (HR = 0.99, 95% CI: 0.55–1.78, *P* = 0.984; *I*^2^ = 0.0%, *P*_heterogeneity_ = 0.853; Figure 4, Table 2). One study reported progression-free survival (PFS) in HPV-18 positivity (23), and an association was observed between HPV-18 positivity and PFS to CC (HR = 2.66, 95% CI: 1.44–4.94, *P* = 0.002; Figure 4, Table 2).

### Sensitivity Analyses

**Figure 5:**
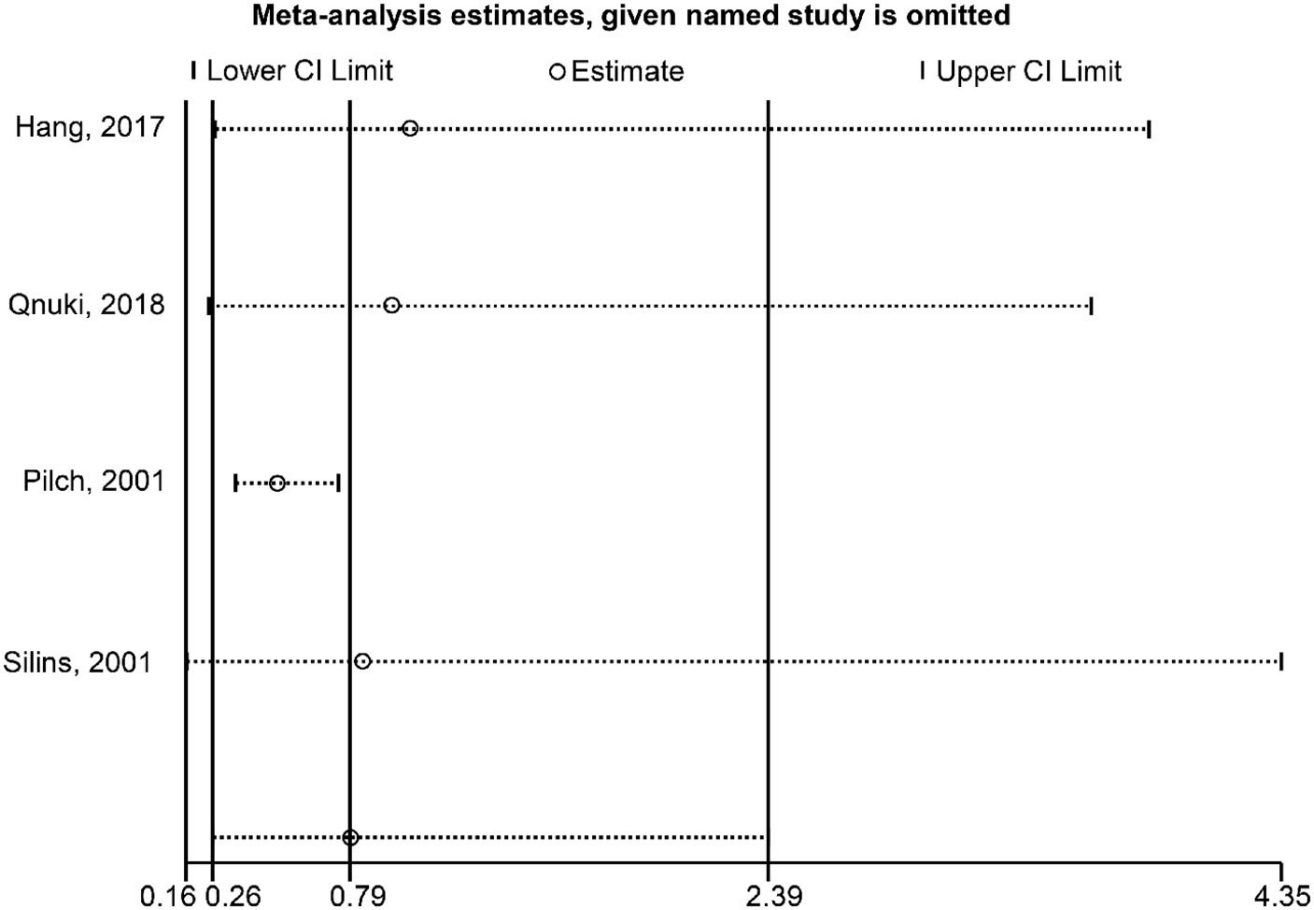
Sensitivity analysis of overall survival comparing the human papillomavirus subtype 16 (HPV-16) positive vs. negative groups.

**Figure 6:**
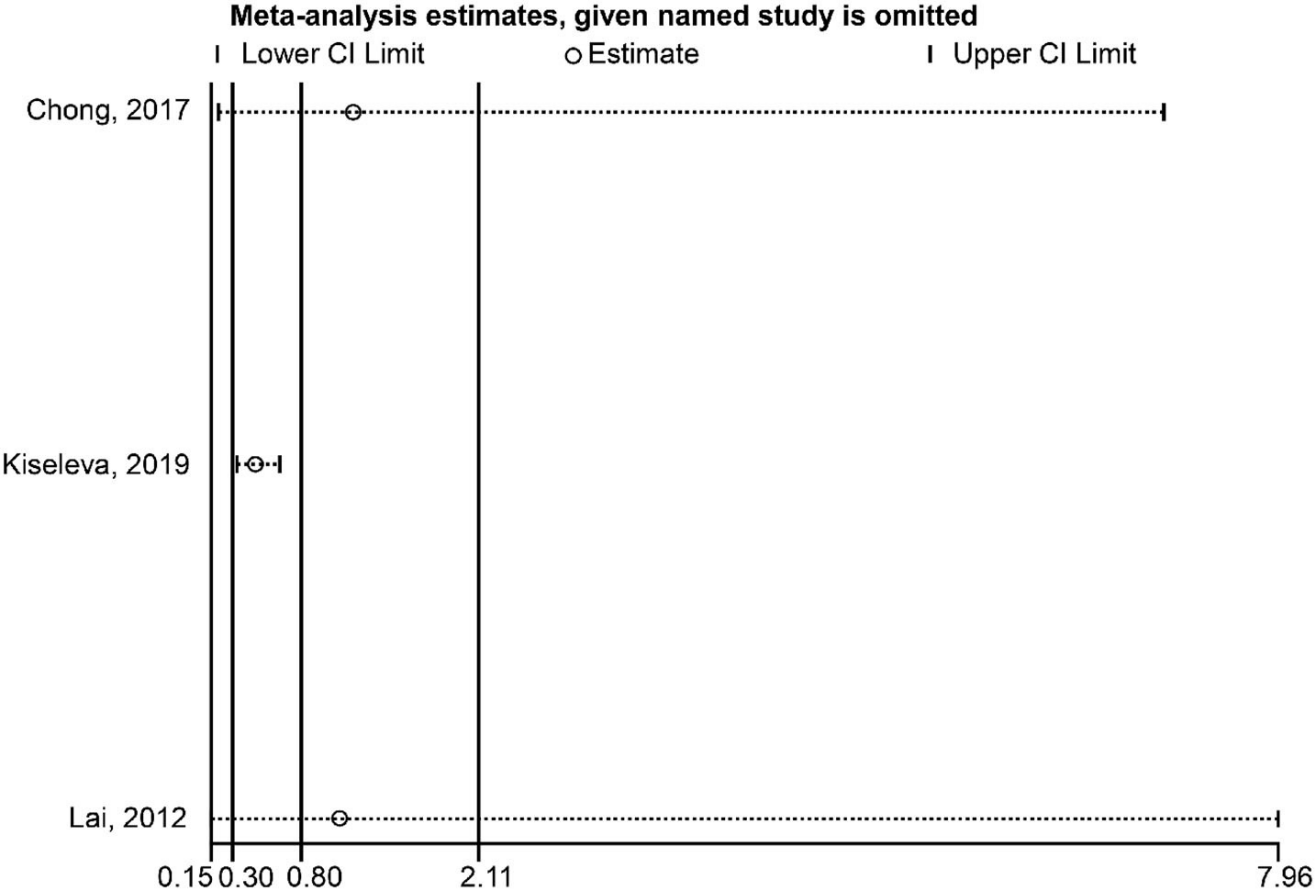
Sensitivity analysis of disease-free survival comparing the human papillomavirus subtype 16 (HPV-16) positive vs. negative groups.

Regarding the association between HPV-16 and OS, the sensitivity analysis showed that omitting Pilch et al. (17) affected the conclusion (Figure 5). Regarding the association between HPV-16 and DFS, the sensitivity analysis showed that omitting Kiseleva et al. (25) affected the conclusion (Figure 6).

## Discussion

The literature reports conflicting results regarding the effect of HPV-16/18 positivity on CC prognosis. Therefore, this meta-analysis aimed to examine the effect of HPV-16/18 positivity on the prognosis of patients with CC. The results suggest that the presence of HPV-16 and HPV-18 positivity appears to have no significant association with prognosis in CC in either OS or PFS. The presence of HPV-16 or HPV-18 positivity has no significant association with the prognosis of CC (either OS or PFS). This is in contradiction to the aggressive feature of HPV-16/18-positive lesions during the development of CIN to CC.

In HPV-associated CC, tumorigenesis is driven by the E6 and E7 oncogenes from the viral DNA integrated into the host cells (29), but in HPV-negative CC, tumorigenesis is driven by the intrinsic oncogenes (30), and the two types of CC could be distinct diseases (13). A recent meta-analysis showed that pretreatment HPV DNA positivity in patients with CC was associated with a better prognosis in Mongoloids and Caucasians (13). Similar results were observed for head and neck cancers (14, 15), but these previous meta-analyses did not examine the HPV types.

It is now well-known that the different HPV types differ widely in terms of epidemiology and potential for CIN and CC (31–36). Among them, HPV-16 and HPV-18 are generally considered as being those at the highest risk of CC (31–36). Available studies suggest a positive effect of HPV16/18 positivity on CC outcomes (16, 22, 26), a negative effect (17, 25), or no effect (27, 28). A nationwide study that was not eligible for the present meta-analysis showed that patients with CC positive for a high-risk HPV type had a better prognosis than patients negative for such types (37), but other non-eligible studies also report conflicting results (30, 38–43). Indeed, Cuschieri et al. (39) showed that patients with CC and HPV-16/18 had better survival than those without HPV-16/18. Wang et al. (42) showed that CC caused by both HPV α-7 (which includes HPV-18) and HPV α-8 (which includes HPV-16) had a better prognosis than CC caused by HPV α-7 alone. Dahlgren et al. (43) reported a better prognosis for CC with HPV-16, but Lai et al. (40) reported a worse prognosis for HPV-18. When considering the eligible studies, the present meta-analysis suggests that there is no association between HPV-16/18 positivity and CC outcomes.

The results of the present meta-analysis must be considered in light of its limitations. We failed to conclude the prognostic effect of HPV-16 and HPV-18 in CC because the eligible studies had conflicting results. Of note, a number of studies could not be included because they did not report results specifically for HPV-16 or HPV-18. To our knowledge, there are only a few studies that investigated the prognostic effect of HPV-16 and HPV-18 in patients with CC, and the survival outcomes in each study were reported differently. Nevertheless, the non-eligible studies also had conflicting conclusions. Despite that nine studies were included in the meta-analysis, never more than four studies were analyzed together for a given outcome. One study reported the risk estimates as RR instead of HR. We treated the RR as HR for the analysis purpose, but it could introduce a bias. The nine studies were all observational studies, decreasing the strength of the conclusion, but a randomized control trial is not possible in this context. In addition, false-negatives could not be taken into account because of nonuniform reporting or non-reporting among the included studies. Finally, the risk estimates of survival outcomes were not reported at the same duration after the patients were discharged from the hospital.

In conclusion, the presence of HPV-16 and HPV-18 positivity appears to have no significant association with prognosis in CC in either OS or PFS, but the sensitivity analysis indicated that the study by Pilch et al. (17) has a strong impact on the outcome. Eliminating this study from the analysis would lead to a conclusion of a better prognosis of HPV-16 positivity in CC. Despite the limitations, the present meta-analysis observed different results on the prognostic effect of HPV-16 and HPV-18 among the existing studies. Future studies with larger numbers of patients in different countries and various ethnicities should be encouraged.

## Data Availability Statement

All datasets generated for this study are included in the article/Supplementary Material.

## Author Contributions

XC substantially contributed to conception or design, contributed to acquisition, analysis, or interpretation of data, drafted the manuscript for important content, critically revised the manuscript for important intellectual content, and gave final approval. PZ substantially contributed to conception or design, contributed to acquisition, analysis, or interpretation of data, drafted the manuscript for important content, and critically revised the manuscript for important intellectual content. SC contributed to acquisition, analysis, or interpretation of data. HZ drafted the manuscript for important content. XDC gave final approval. All authors contributed to the article and approved the submitted version.

## Conflict of Interest

The authors declare that the research was conducted in the absence of any commercial or financial relationships that could be construed as a potential conflict of interest.
