# Supplementary material for: Better or Worse? The Independent Prognostic Role of HPV-16 or HPV-18 Positivity in Patients With Cervical Cancer: A Meta-Analysis and Systematic Review
Source: Front Oncol. 2020 Oct 7;10:1733. doi: 10.3389/fonc.2020.01733 (PMC7577117; doi:10.3389/fonc.2020.01733)
Supplement: Supplementary file 1 [file Table_1.DOCX]

**Supplementary Table S1.** NOS criteria for quality of cohort study.

| Study | Representativeness of the exposed cohort | Selection of the non-exposed cohort | Ascertainment of exposure | Demonstration that the outcome of interest was not present at the start of the study | Comparability of cohorts on the basis of the design or analysis | Assessment of outcome | Was follow-up long enough for outcomes to occur | Adequacy of follow up of cohorts | Total quality scores |
| --- | --- | --- | --- | --- | --- | --- | --- | --- | --- |
| Chong, 2017 (1) | ☆ | ☆ | ☆ | ☆ | ☆☆ | ☆ | ☆ | - | 8 |
| Hang, 2017 (2) | ☆ | ☆ | ☆ | ☆ | ☆☆ | ☆ | ☆ | - | 8 |
| Kang, 2010 (3) | ☆ | ☆ | ☆ | ☆ | ☆☆ | ☆ | ☆ | - | 8 |
| Kim, 2011 (4) | ☆ | ☆ | ☆ | ☆ | ☆☆ | - | ☆ | - | 7 |
| Kiseleva, 2019 (5) | ☆ | ☆ | ☆ | ☆ | ☆☆ | ☆ | ☆ | - | 8 |
| Lai, 2012 (6) | ☆ | ☆ | ☆ | ☆ | ☆☆ | - | ☆ | - | 7 |
| Onuki, 2018 (7) | ☆ | ☆ | ☆ | ☆ | ☆☆ | - | ☆ | - | 7 |
| Pilch, 2001 (8) | ☆ | ☆ | ☆ | ☆ | ☆☆ | ☆ | ☆ | ☆ | 9 |
| Silins, 2001 (9) | ☆ | ☆ | ☆ | ☆ | ☆☆ | - | ☆ | - | 7 |

**References**

1. Chong GO, Lee YH, Han HS, Lee HJ, Park JY, Hong DG, et al. Prognostic value of pre-treatment human papilloma virus DNA status in cervical cancer. *Gynecologic oncology* (2018) 148(1):97-102. doi: 10.1016/j.ygyno.2017.11.003. PubMed PMID: 29153540.

2. Hang D, Jia M, Ma H, Zhou J, Feng X, Lyu Z, et al. Independent prognostic role of human papillomavirus genotype in cervical cancer. *BMC infectious diseases* (2017) 17(1):391. doi: 10.1186/s12879-017-2465-y. PubMed PMID: 28583086; PubMed Central PMCID: PMC5460478.

3. Kang WD, Kim CH, Cho MK, Kim JW, Cho HY, Kim YH, et al. HPV-18 is a poor prognostic factor, unlike the HPV viral load, in patients with stage IB-IIA cervical cancer undergoing radical hysterectomy. *Gynecologic oncology* (2011) 121(3):546-50. doi: 10.1016/j.ygyno.2011.01.015. PubMed PMID: 21334052.

4. Kim JY, Nam BH, Lee JA. Is human papillomavirus genotype an influencing factor on radiotherapy outcome? Ambiguity caused by an association of HPV 18 genotype and adenocarcinoma histology. *Journal of gynecologic oncology* (2011) 22(1):32-8. doi: 10.3802/jgo.2011.22.1.32. PubMed PMID: 21607093; PubMed Central PMCID: PMC3097332.

5. Kiseleva VI, Mkrtchyan LS, Ivanov SA, Lyubina LV, Bezyaeva GP, Panarina LV, et al. The Presence of Human Papillomavirus DNA Integration is Associated with Poor Clinical Results in Patients with Third-Stage Cervical Cancer. *Bulletin of experimental biology and medicine* (2019) 168(1):87-91. doi: 10.1007/s10517-019-04654-2. PubMed PMID: 31768781.

6. Lai CH, Chou HH, Chang CJ, Wang CC, Hsueh S, Huang YT, et al. Clinical implications of human papillomavirus genotype in cervical adeno-adenosquamous carcinoma. *European journal of cancer* (2013) 49(3):633-41. doi: 10.1016/j.ejca.2012.09.008. PubMed PMID: 23031554.

7. Onuki M, Matsumoto K, Tenjimbayashi Y, Tasaka N, Akiyama A, Sakurai M, et al. Human papillomavirus genotype and prognosis of cervical cancer: Favorable survival of patients with HPV16-positive tumors. *Papillomavirus research* (2018) 6:41-5. doi: 10.1016/j.pvr.2018.10.005. PubMed PMID: 30347290; PubMed Central PMCID: PMC6218653.

8. Pilch H, Gunzel S, Schaffer U, Tanner B, Brockerhoff P, Maeurer M, et al. Human papillomavirus (HPV) DNA in primary cervical cancer and in cancer free pelvic lymph nodes--correlation with clinico-pathological parameters and prognostic significance. *Zentralblatt fur Gynakologie* (2001) 123(2):91-101. doi: 10.1055/s-2001-12411. PubMed PMID: 11265139.

9. Silins I, Avall-Lundqvist E, Tadesse A, Jansen KU, Stendahl U, Lenner P, et al. Evaluation of antibodies to human papillomavirus as prognostic markers in cervical cancer patients. *Gynecologic oncology* (2002) 85(2):333-8. doi: 10.1006/gyno.2002.6628. PubMed PMID: 11972397.
